# How to discuss gene therapy for haemophilia? A patient and physician perspective

**DOI:** 10.1111/hae.13769

**Published:** 2019-05-21

**Authors:** Wolfgang Miesbach, Brian O’Mahony, Nigel S. Key, Mike Makris

**Affiliations:** ^1^ Department of Haemostaseology and Haemophilia Centre, Medical Clinic 2, Institute of Transfusion Medicine University Hospital Frankfurt Frankfurt Germany; ^2^ Chief Executive Irish Haemophilia Society Dublin Ireland; ^3^ Trinity College Dublin Ireland; ^4^ Division of Hematology/Oncology, Department of Medicine University of North Carolina Chapel Hill North Carolina; ^5^ Sheffield Haemophilia and Thrombosis Centre Royal Hallamshire Hospital Sheffield UK; ^6^ Department of Infection Immunity and Cardiovascular Disease University of Sheffield Sheffield UK

**Keywords:** Adeno‐associated virus, factor IX, factor VIII, gene therapy, haemophilia

## Abstract

Gene therapy has the potential to revolutionise treatment for patients with haemophilia and is close to entering clinical practice. While factor concentrates have improved outcomes, individuals still face a lifetime of injections, pain, progressive joint damage, the potential for inhibitor development and impaired quality of life. Recently published studies in adeno‐associated viral (AAV) vector‐mediated gene therapy have demonstrated improvement in endogenous factor levels over sustained periods, significant reduction in annualised bleed rates, lower exogenous factor usage and thus far a positive safety profile. In making the shared decision to proceed with gene therapy for haemophilia, physicians should make it clear that research is ongoing and that there are remaining evidence gaps, such as long‐term safety profiles and duration of treatment effect. The eligibility criteria for gene therapy trials mean that key patient groups may be excluded, eg children/adolescents, those with liver or kidney dysfunction and those with a prior history of factor inhibitors or pre‐existing neutralising AAV antibodies. Gene therapy offers a life‐changing opportunity for patients to reduce their bleeding risk while also reducing or abrogating the need for exogenous factor administration. Given the expanding evidence base, both physicians and patients will need sources of clear and reliable information to be able to discuss and judge the risks and benefits of treatment.

## 1 INTRODUCTION

### 1.1 Gene therapy for haemophilia

Gene therapy (GT) for haemophilia is being evaluated for its potential to provide long‐term, potentially curative treatment for people with haemophilia (PWH) by increasing endogenous clotting factor activity. This approach could replace the current standard of care, namely exogenous factor replacement that has undergone significant improvements over the last few decades but remains suboptimal in terms of preserving joint and overall health and is associated with a significant quality of life (QoL) burden. While GT has the potential to improve physical health and overall QoL, clinical experience is still relatively limited. This article provides perspectives from a haemophilia patient advocate, with personal experience of the disease, as well as physicians involved in clinical care regarding where GT might address unmet needs and mitigate the disease burden for PWH. It should be noted that due to limitations in the available evidence, some of the expert perspectives expressed in the manuscript will necessarily reflect personal experience and are yet unsupported by published peer‐reviewed studies.

### 1.2 The burden of haemophilia

The introduction of clotting factor therapy in the 1960s and 1970s transformed life expectancy for severe haemophilia from under 30 years to near normal.^1^ The contamination of clotting factor concentrates (CFCs) prepared from pooled plasma with HIV and hepatitis viruses, however, blighted many lives.^2^ Safety improved with the introduction of effective viral inactivation measures followed by recombinant DNA technology in the 1980s.^2^, ^3^ Since then, CFCs have evolved with the development of extended half‐life (EHL) versions that improve the QoL by reducing dosing frequency^4^, ^5^ and increase protection by enabling higher trough levels. Despite this, haemophilia continues to impose multiple complications including joint damage, functional impairment, acute and chronic pain, mental health/anxiety issues, reduced QoL, as well as impaired social participation, reduced educational attainment and diminished work productivity (Table 1).

**Table 1:** Burden of haemophilia

Burden	Cause
Joint damage	Can result in chronic pain, disability and joint deformity at an early age 1, 54, 55
Poor health‐related quality of life	Closely linked to the extent of joint damage 54
Functional impairment	More likely to suffer from arthropathy/arthritis, more likely to require knee/hip replacement compared with the general population.^1^, ^56^ Poor mobility, self‐care issues, and inability to perform usual daily activities 57, 58
Social isolation	Inability to participate in social or sporting activities 59
Pain	Higher pain levels and functional impairment associated with anxiety, depression and unemployment.^60^, ^61^ Pain/discomfort is an area where most individuals report experiencing ‘extreme’ issues.^54^ Individuals may experience anger and frustration due to the pain, inconvenience and erratic nature of bleeds 62
Psychological	Anxiety/depression are the areas where most individuals report experiencing ‘extreme’ issues 54
Personal productivity	Adverse impact on educational achievement and work productivity due to absence and difficulties due to functional impairments and pain 57, 63, 64

### 1.3 Unmet needs in haemophilia treatment

The limitations of current options highlight the need for less burdensome and more cost‐effective treatment that limits the longer‐term complications experienced by PWH (Table 2). Preliminary evidence in haemophilia A and B indicates that GT may offer the potential to address these limitations.

**Table 2:** Current unmet needs in haemophilia treatment

Unmet need	Impact
Treatment convenience	Lifetime treatment, frequent injections.^65^, ^66^ Prophylaxis is time‐consuming, contributing to poor adherence 67
Joint damage despite factor prophylaxis	Indicates that prophylaxis is failing to control some subclinical bleeding 55, 68
Inhibitor development	Occurs in approximately one‐third of patients with severe haemophilia A and <5% of those with haemophilia B and increases treatment cost and morbidity risks 69
High lifetime‐treatment costs	High factor concentrate costs,^1^, ^70^, ^71^, ^72^ means availability of factor prophylaxis is limited in many countries
Pain	See Table 1
Limits on activity and social participation	See Table 1

## 2 WHAT IS GENE THERAPY

GT refers to the treatment of a disease through introducing a functional copy of a disease‐causing gene, inactivation of the gene's effects through addition of novel or modified genes, or editing of a host gene to correct a congenital mutation.^6^ GT strategies that are currently approved, or approaching approval, are largely aimed at treating diseases that are caused by a defect in a single gene, such as haemophilia, lipid disorders, retinal diseases and spinal muscular atrophy. The most common way to introduce therapeutic genes is via a viral vector. Unlike earlier approaches using adenoviral and retroviral vectors which insert the transgene into the genome of the host,^7^ recombinant adeno‐associated virus (rAAV) vectors generally remain in the nucleus of the transduced cell in non‐integrated episomal concatemer form (vector DNA linked head to tail in a circular form), with only rare, random integrations into host DNA.^8^ This reduces the potential for genotoxicity with rAAV vectors when compared to insertional vectors. While such rare integrations do not appear to have been associated with clinical sequelae in animal models or clinical studies, it should be remembered that the large number of vector genomes (vg) administered during a typical GT treatment means that there is the potential for a large numbers of random integration events.

Safety concerns regarding initial GT studies using insertional vectors in the 1990s included the death of a patient following adenoviral therapy for ornithine transcarbamylase and multiple leukaemia cases following a retroviral therapy for severe combined immunodeficiency and Wiskott‐Aldrich syndrome.^9^, ^10^, ^11^, ^12^, ^13^ More recently, rAAV vectors have been used most commonly as they effectively transduce target cells but have a lower risk of immunogenicity compared with adenoviral vectors and have a low risk of genotoxicity versus insertional vectors.^14^ AAV is internalised into target cells by binding to specific cell‐surface receptors and is trafficked to the nucleus. In the nucleus, the AAV uncoats by releasing viral DNA from the capsid and the vector transgene is transcribed and expressed.^8^

### 2.1 What evidence supports gene therapy for haemophilia?

#### 2.1.1 Efficacy

Several publications have provided in‐depth reviews of the efficacy and safety of GT in haemophilia,^15^, ^16^, ^17^ so only brief coverage of therapies in active development is included here.

##### Haemophilia A

There are several developmental GT trials for haemophilia A including valoctocogene roxaparvovec (BNM 270, Phase 3) at up to 6 × 10^13^ vg/kg, SPK‐8011 (Phase 1/2) at up to 2 × 10^12^ vg/kg, BAX 888 (Phase 1/2) at an unstated dose, AAV2/8‐HLP‐FVIII‐V3 (Phase 1) at up to 6 × 10^12^ vg/kg, SB‐525 (AAV‐FVIII, Phase 1/2) at an unstated dose and BAY2599023 (AAV‐human B domain‐depleted FVIII) at an unstated dose.^18^, ^19^ In a trial of valoctocogene roxaparvovec (AAV5/B domain‐depleted hFVIII) in nine men, the seven participants in the high dose group (6 × 10^13^ vg/kg) achieved FVIII values above the 5 IU/dL cut‐off for mild haemophilia for up to 52 weeks (range 19‐164 IU/dL at 52 weeks).^18^ These levels were associated with a reduction in median annualised bleed rate from 16 to 1 with cessation in the need for FVIII replacement by week 22 (Table 3A).^18^.

**Table 3:** Trials of AAV gene therapy for haemophilia listed as being active on clinicaltrials.gov

A) Haemophilia A								
	AAV2/8‐HLP‐FVIII‐V3^73^	SB‐525^47^	BAX 888^47^	Valoctocogene roxaparvovec^18^	Valoctocogene roxaparvovec^47^	Valoctocogene roxaparvovec^47^	BAY2599023 (DTX201)^47^	SPK‐8011^74^
Study details								
Name/description	GO‐8	Dose ranging study	Dose ranging & safety	Phase 1/2	Pre‐existing anti‐AAV5 antibodies	Phase 3	BAY2599023 (DTX201)	rAAV with improved liver tropism
NCT number	NCT03001830	NCT03061201	NCT03370172	NCT03569891	NCT03520712	NCT03392974	NCT03588299	NCT03003533
Status	Recruiting	Recruiting	Recruiting	Active, not recruiting	Enrolling	Enrolling	Recruiting	Recruiting
Therapy								
Vector/transgene	AAV2/8‐HLP‐ FVIII‐V	AAV2/6‐hFVIII	AAV8‐ B Domain‐deleted factor VIII	AAV5/B domain‐depleted hFVIII	AAV5/B domain‐depleted hFVIII	AAV5/B domain‐depleted hFVIII	Not stated	Not stated
Study characteristics								
Number of participants	18	20	10	9	10	40	18	12
Length of follow‐up	Up to 15 y	Up to 3 y	Up to 3 y	1 y	Up to 5 y	Up to 5 y	Up to 5 y	1 y
Design	Phase 1	Phase 1/2	Phase 1/2	Phase 1/2	Phase 1/2	Phase 3	Phase 1/2	Phase 1/2
Dose, vg/kg	6 × 10^11^ 2 × 10^12^ 6 × 10^12^	Not stated	Not stated	6 × 10^12 ^(Co. 1, n = 1) 2×10^13 ^(Co. 2, n = 1) 6 × 10^13^ (Co. 3, n = 7)	6 × 10^13^	4 × 10^13^	Not stated	6 × 10^11^ (n = 2) 1 × 10^12^ (n = 3) 2 × 10^12 ^(n = 7)
Baseline characteristics								
FVIII and FIX activity, IU/dL or %	<1	<1	<1	<1	<1	<1	<1	<1
Efficacy								
Endogenous FIX or FVIII activity	>5 IU/dL (7, 6 and 69 IU/dL) at 6 weeks, n = 3	TBC	TBC	<1 IU/dL (Co. 1) 2 IU/dL (Co. 2) 19‐164 IU/dL (Co. 3)	TBC	TBC	TBC	13%‐49%
Reduction in annualised FIX or FVIII use, %	TBC	TBC	TBC	9 (Co. 1) 88 (Co. 2) 99 (Co. 3)	TBC	TBC	TBC	97
Reduction in annualised bleeds, %	TBC	TBC	TBC	Not reported (Co. 1) NA^c^ (Co. 2) 88 (Co. 3)	TBC	TBC	TBC	97
Safety								
Serious AE^d^	TBC	TBC	TBC	Progression of chronic arthropathy	TBC	TBC	TBC	Grade 2 ALT elevation, FVIII decline, IFN‐γ production
Treatment‐related AE	No Grade III or greater AEs	TBC	TBC	ALT elevations, arthralgia, back pain, fatigue, productive cough	TBC	TBC	TBC	TBC
ALT elevations leading to reduction/loss of FIX activity	Elevations in 2/3, no loss of FIX activity	TBC	TBC	1/8	TBC	TBC	TBC	2/12

B) Haemophilia B: AAV gene therapy with wild‐type FIX			
	AMT‐060^20^	scAAV2/8‐LP1‐hFIXc^21^, ^22^, ^a^	AskBio009
Study details			
Name/description	Phase 1/2	Phase 1	Phase 1/2
NCT number	NCT02396342	NCT00979238	NCT01687608
Status	Active, not recruiting	Active, not recruiting	Active, not recruiting
Therapy			
Vector/transgene	AAV5/codon‐optimised wild‐type hFIX	AAV8/codon‐optimised wild‐type hFIX	AAV8/FIX
Study characteristics			
Number of participants	10	14	30
Length of follow‐up	1 y (5‐year follow‐up in progress)	Up to 3 y	Up to 5 y
Design	Phase 1/2	Phase 1	Phase 1/2
Dose, vg/kg	5 × 10^12 ^(Co. 1, n = 5) 2 × 10^13 ^(Co. 2, n = 5)	2×10^11^(Co. 1, n = 2) 6 × 10^11 ^(Co. 2, n = 2) 2 × 10^12^ (Co. 3, n = 2)	Not stated
Baseline characteristics			
FVIII and FIX activity, IU/dL	<2^b^	<1	≤2
Efficacy			
Endogenous FIX or FVIII activity	4.4 IU/dL (Co. 1) 6.9 IU/dL (Co. 2)	1.8% (Co. 1) 2.5% (Co. 2) 5.1% (Co. 3)	TBC
Reduction in annualised FIX or FVIII use, %	81 (Co. 1) 73 (Co. 2)	92 (overall) 96 (Co. 3)	TBC
Reduction in annualised bleeds, %	53 (Co. 1) 70 (Co. 2)	90 (overall) 94 (Co. 3)	TBC
Safety			
Serious AE^d^	ALT elevation of mild^e^severity (n = 2), self‐limiting fever (n = 1)	No	TBC
Treatment‐related AE	ALT elevations (3/10), pyrexia (3/10), anxiety (2/10), palpitations (1/10), headache (1/10), prostatitis (1/10), rash (1/10)	ALT elevations (4/6 in Co. 3), lethargy, anaemia	TBC
ALT elevations leading to reduction/loss of FIX activity	0/3	4/4	TBC

C) Haemophilia B: AAV gene therapy with Padua FIX variant				
	SPK‐9001^23^	AAV5‐hFIXco‐Padua (AMT‐061)^75^	AAV5‐hFIXco‐Padua (AMT‐061)^76^	FLT180a^24^
Study details				
Name/description	Phase 1/2a Long‐term safety & efficacy	Phase 2b Dose confirmation	Phase 3 HOPE‐B	Phase 1/2 FIX‐GT
NCT number	NCT03307980	NCT03489291	NCT03569891	NCT03369444
Status	Active, not recruiting	Recruiting	Recruiting	Recruiting
Therapy				
Vector/transgene	AAV/FIX Padua variant	AAV5/FIX Padua variant	AAV5/FIX Padua variant	AAV/FIX Lys‐Arg change at position 301 variant
Study characteristics				
No. participants	20	3	56	18
Length of follow‐up	FIX activity (5 y) ABR (5 y) FIX replacement (5 y) AEs (5 y)	FIX activity (6 wk) ABR (1 y) FIX replacement (1 y) AEs (5 y)	FIX activity (26 wk) ABR (1 y) FIX replacement (1 y) AEs (5 y)	26 wk
Design	Phase 2	Phase 3	Phase 3	Phase 1/2
Dose, vg/kg	5 × 10^11^	2 × 10^13^	2 × 10^13^	4.5 × 10^11^ (n = 2, first enrolled patients)
Baseline characteristics				
FVIII and FIX activity, IU/dL	<2	<2	<2	<1 or 1‐2
Efficacy				
Endogenous FIX or FVIII activity	33.7%	31%	TBC	>40%
Reduction in annualised FIX or FVIII use, %	91‐100	TBC	TBC	100
Reduction in annualised bleeds, %	96	TBC	TBC	No spontaneous bleeds, 1 traumatic bleed
Safety				
Serious AE^d^	No	TBC	TBC	None
Treatment‐related AE	ALT elevations	TBC	TBC	Not reported
ALT elevations leading to reduction/loss of FIX activity	1/2	TBC	TBC	0/0

AE, adverse event; ALT, alanine aminotransferase; Co., cohort; hFIX, human Factor IX; TBC, to be confirmed; vg, vector genomes.

a Results reflect long‐term follow‐up.^22^

b With severe bleeding phenotype.

c 3 and 11 bleeds pre‐ and post‐therapy, respectively.

d SAE was defined as any untoward medical occurrence that at any dose: results in death; is life‐threatening; requires in‐patient hospitalisation or prolongation of existing hospitalisation; results in persistent or significant disability or incapacity; is a congenital anomaly or birth defect; or is judged medically important by the investigator.

e The severity of AEs was defined as: Mild: Awareness of symptoms, sign, illness or event that is easily tolerated; Moderate: Discomfort sufficient to cause interference with usual activity; or Severe: Incapacitating with inability to work or undertake further normal activities. These tables included trials that were listed as ‘Active’ on https://clinicaltrials.gov on the 18 December 2018. Therefore, trials that were listed as ‘Terminated’ at that time, or those that were not listed on ClinicalTrial.gov were not included. The trial of SB‐FIX, a zinc finger nuclease that is delivered by an AAV vector and which inserts a functional FIX gene into hepatocytes, was not included in the table.

##### Haemophilia B

For several haemophilia B, AAV‐based GT is being developed including two currently enrolling for a phase 3 study (AMT‐061, SPK‐9001), one with long‐term follow‐up from Phase 1/2 (scAAV2/8‐LP1‐hFIXc), and one in early Phase 1/2 (FLT180a) that are starting to present early data on small numbers of participants with limited follow‐up.^20^, ^21^, ^22^, ^23^, ^24^ In addition, SB‐FIX, a zinc finger nuclease that inserts a functional FIX gene into hepatocytes, is recruiting for phase 1 (https://clinicaltrials.gov).

Vector‐mediated GT in haemophilia B has demonstrated that it is possible to convert patients with severe disease (<1% FIX activity) to a ‘mild’ phenotype, that is endogenous FIX levels of 5% or more with vectors that carry wild‐type FIX such as AMT‐060 or scAAV2/8‐LP1‐hFIXc (Table 3B).^20^, ^21^, ^22^, ^23^ In addition, GT utilising wild‐type FIX is associated with the cessation of factor prophylaxis in most participants, the reduction in exogenous factor usage by 73%‐96% and a reduction in annualised bleed rates of between 70% and 94% in those groups who achieved mean FIX activity >5%.^20^, ^23^

In order to increase FIX expression, several groups have used other variants, such as the naturally occurring FIX Padua variant (eg SPK‐9001 and AMT‐061) and a variant with a novel lysine to arginine substitution at position 301 (FLT180a), which enhance FIX activity (Table 3C). With these approaches, FIX activities in the range of 30% to >40% have been reported along with reductions in annualised bleeds and exogenous FIX use of approximately 90% to 100%. In addition, these variants may allow a lower dose of GT to be used, which may be useful if vector dose is a factor in the development of capsid‐specific immune responses.

#### 2.1.2 Safety

The safety profile of AAV vectors reflects the fact they are related to naturally occurring AAV, which are generally non‐pathogenic in humans. As has been discussed, recombinant AAV only rarely integrates into host DNA,^8^ minimising the potential for genotoxicity.^7^ Based on relatively limited data from 35 participants, one of the main adverse events that was observed in 17 of 35 participants (48.6%) across all trials was transient alanine aminotransferase (ALT) elevations (Table 3), which has also been observed in previous GT trials utilising intramuscular injection.^25^ While ALT elevations are not a safety issue per se, as these events were generally asymptomatic and were treated with a course of corticosteroids, in some cases they have been associated with a reduction in factor activity (Table 3).^18^, ^20^, ^21^, ^22^, ^23^ However ALT elevations, along with worse than expected FIX activity,^26^ resulted in the discontinuation of AAVrh10FIX (DTX101) a candidate therapy for haemophilia B. In haemophilia A, ALT elevations did not appear to reflect the dose of vector administered with AAV8‐HLP‐hFVIII‐V at 6 × 10^11^ vg/kg (n = 1 of 1) and 2 × 10^12^ vg/kg (n = 1 of 2), or with valoctocogene roxaparvovec (6 × 10^12^ to 6 × 10^13^ vg/kg). To deal with ALT elevations, at least three trials including the valoctocogene roxaparvovec and SPK‐8011 trials in haemophilia A and the FLT180a trial in haemophilia B have used prophylactic steroid treatment.^18^, ^24^, ^27^ Other treatment‐emergent adverse events associated with GT include lethargy/fatigue,^18^, ^22^ anaemia 21 and back pain (Table 3B).^18^ Long‐term safety is uncertain as the length of follow‐up in published studies is generally from 1 year up to a maximum of eight years.^28^, ^29^ There are, however, positive safety reports from longer‐term follow‐up in animals.^30^.

### 2.2 Unmet needs

Recent years have seen a major expansion in treatment options with the wider availability of EHL CFCs. EHL factors have allowed a greater bleed protection by enabling higher trough levels to be achieved and have reduced the frequency of intravenous (IV) infusions. However, PWH treated with EHL CFCs continues to be dependent on regular injections and must always be cognisant of the peaks and troughs of their factor levels in relation to their activity. It is also clear that the same disparity of access which we have seen with standard half‐life CFCs continues with EHL factors. The 2018 European Haemophilia Consortium (EHC) survey of 40 European countries,^31^ identified only 10 countries that always, or sometimes, had access to EHL CFCs, and there was practically no access in Eastern or Central European countries.

Another advance in treatment has been the development of humanised bispecific antibody technology, which by binding to both activated FIX and factor X can mimic the action of FVIII (emicizumab). Subcutaneous (SC) emicizumab therapy for FVIII deficiency with and without inhibitors has been licenced by the FDA,^32^ which will offer a degree of freedom from fluctuating factor levels by conferring a constant level of protection while also removing the burden of IV infusion.^33^, ^34^ The level of protection conferred appears to be in the range which will prevent most bleeds, but does not confer a normal or near‐normal level of protection, so treatment for breakthrough bleeds and surgery with FVIII clotting factor will continue to be required.

Expectations of GT have changed significantly over the previous five years as we have seen FIX expression increase from a modest 4.4%‐7% in early trials 20, 21, 22 to 33% more recently,^23^ with the current hope being sustained expression of factor level in the normal range. For haemophilia A, the valoctocogene roxaparvovec from Biomarin has demonstrated expression in the normal range (>50 IU/dL) at 52 weeks in 6 of the seven participants in the highest‐dose group and monitoring continues to assess the duration of expression.^18^ Crucially, normal factor levels should be sufficient to free PWH from any requirement for treatment with factor concentrates in all situations, including surgery.

CoreHEM used a modified Delphi decision‐making process with a group of 49 experts including PWH, clinicians, researchers, regulators, health technology assessors, payers and drug developers to identify outcomes of most importance to PWH.^35^ CoreHEM identified factor level, duration of expression of factor level, impact on chronic pain, healthcare resource utilisation, impact on mental health and frequency of bleeds as the key outcomes. A factor level in the normal range should transform the QoL of PWH. From a patient perspective, the duration of expression should ideally be lifelong but, if not, should be sustained over many years. With the current technology, re‐treatment with the same vector is not possible, and in any case the economics of GT may not allow this. Chronic pain impacts most PWH due to a combination of target joints, pre‐existing haemophilic arthropathy and subclinical bleeds. Anecdotally, there have been reports from people treated with EHL FIX or SC therapy for FVIII of a significant decrease in joint aches and pains. This may reflect higher trough levels, especially with EHL FIX, or higher equivalent level of protection conferred by SC therapy for FVIII leading to a significant decrease in subclinical bleeds. Therefore, it will be of great interest to assess the long‐term impact of GT on acute and chronic pain as well as the arthropathy, although, for those who already have end‐stage arthropathy, the impact may be minimal.

Gene therapy with a factor expression in the normal range would free PWH from their mental burden and may lead to a real reduction in the levels of anxiety and depression.^36^ With greater levels of protection, the frequency of bleeds should decrease even in the presence of higher levels of physical activity. Freedom to carry out normal everyday activities, taken for granted by those without haemophilia, such as walking, running, cycling, swimming and potentially riskier sports participation would all become more attainable.

## 3 HOW TO DISCUSS GENE THERAPY: PHYSICIAN AND PATIENT PERSPECTIVES ON EFFICACY AND SAFETY

### 3.1 Efficacy

From a physician perspective, it will be key to manage patient expectations of GT, particularly in the early days following treatment. GT has demonstrated the ability to convert individuals from a ‘severe’ to ‘mild’ phenotype in terms of endogenous factor activity; however, treated adults are likely to have a legacy of joint damage that may increase bleed risk even in the post‐GT setting with normal or near‐normal endogenous factor activity. During the initial post‐GT period, and depending on the attained factor level, physicians should advise individuals that GT should not be considered as ‘cured’, that they may continue to require clinical monitoring despite having ‘mild’ haemophilia, and that any increase in physical activity should be undertaken cautiously. In trials to date there have been initial indications that the bleed risk diminishes with longer length of follow‐up after GT,^18^, ^20^ so it will be of interest to determine whether the presence of stable factor levels over the longer term can induce clinical improvements in target joints and therefore reduce bleed risk.^15^ Changes in how individuals manage their haemophilia may cause stress or anxiety, so emotional support may be needed. Another aspect that should become clearer with increasing experience is whether potential determinants of responses to GT such as the extent of joint damage, presence of neutralising antibodies, potential markers of the likelihood of T‐cell‐mediated immune responses, or other currently unknown prognostic factors can be identified.

Patients are likely to be interested in how long they can expect the benefits of GT to persist. Within the limited follow‐up of current trials, GT for haemophilia B has resulted in stable FIX expression for up to eight years,^28^, ^29^ however, the longer‐term durability of expression remains to be determined. Given that recombinant AAV does not generally integrate into host genomes, levels of transduction are expected to fall as cells turnover and die.^8^, ^37^ Under normal conditions, most hepatocytes are in a quiescent state with <1%‐2% undergoing turnover at any time.^38^, ^39^ While there is some uncertainty due to limited data, each non‐resting hepatocyte has an estimated lifespan of 200‐300 days, so it is likely that the documented stable FIX levels reflect low hepatocyte turnover.^29^, ^38^ Patients should also be made aware of the fact that the apparent lack of clinically relevant integration of the AAV vector also means that any benefits from GT will not be passed on to children. Therefore, following the initial meeting with their physician, individuals should write down any questions they have and ensure they are answered. They should decide what outcomes they would consider to be acceptable, in terms of factor activity, duration of factor expression and the potential level of bleed reduction, while appreciating that there are still uncertainties in terms of the level and duration of factor expression. Given that GT is still in the investigational stage, however, individuals should be prepared for the possibility of a poor outcome (such as low expression, no expression or early loss of expression).

From a patient perspective, resource utilisation is important, particularly in those countries in which treatment costs are borne by the individual or treatment can be refused by health insurance companies. They will reasonably expect the best treatment from their providers and strong advocacy from their representative patient organisations. For healthcare provided by a national health service or national insurance model, it is likely that an amortisation payment model, in which the initial treatment cost is spread by making payments over several years, may become the preferred model. This would be cost‐effective, would not have an enormous budget impact in year one and could include an element of risk sharing if the continued payment was linked to continued factor expression at a defined level. It may be the case that GT becomes a more attractive option even for developing and emerging countries where the current high‐lifetime costs of CFC treatment are not seen as sustainable.

### 3.2 Safety

GT is a relatively new technology that is starting to enter clinical practice. As is clear from the previous section, the current clinical evidence for GT in haemophilia reflects limited follow‐up in a relatively small number of PWH.^18^, ^20^, ^21^, ^22^, ^23^ Thus, when addressing questions about GT, physicians should be clear that there are areas of uncertainty, such as longer‐term efficacy and safety, for which only further clinical experience will provide answers.

A key worry is GT safety, particularly due to the serious safety concerns in the early trials using integrating vectors.^40^ The majority of current trials use AAV vectors, which are much less likely to integrate into the host genome or cause malignant transformation compared with integrating vectors. As discussed in the previous section, no major safety issues with AAV‐mediated GT have been identified, although the current follow‐up periods are relatively short. The normal range of FIX and FVIII in people without haemophilia is 50% to 150% of normal, however, FIX values >125% and FVIII values >100% may be associated with increased thrombogenicity.^40^, ^41^, ^42^ There does not appear to be an issue in FIX gene transfer, which is associated with FIX activity at the lower range of normal. In contrast, FVIII gene transfer has been associated with FVIII activity >150% of normal in some participants, which was not associated with thrombotic events based on a small number of participants with limited follow‐up^18^; however, it makes sense to avoid inducing supraphysiologic levels of FVIII.

From a practical perspective, vector DNA is detectable in bodily fluids for variable periods ranging from 2‐28 weeks in urine, 4‐52 weeks in saliva, 4‐56 weeks in semen, 16‐52 weeks in faeces, and from 4 weeks to >1 year in blood.^20^ The detection of vector genome fragments does not indicate infectious risk as the test does not distinguish between infectious vector particles, and free, episomal, or integrated DNA. Importantly, recombinant AAV vectors are designed so that they are unable to replicate. In non‐human primates, while vector genome sequences were identified in different cell populations and tissues for up to 18 months, infectious vector particles were rapidly cleared within 72 hours.^43^ In other animal studies, sperm cells appear to be refractory to AAV transduction, lessening the risks of vertical transmission.^44^ However, while the risk of third‐party infection is limited, physicians should recommend barrier contraception for up to 12 months as a precaution.

From a patient perspective concerns may include the risk of mutagenesis due to vectors insertional events (Table 4). Other concerns may include vector shedding and the risk of infecting family members and close contacts. Patients may also be worried about whether GT may increase the risk of inhibitor induction. There may also be anxiety related to potential trade‐offs between increasing the vector dose, the level of factor activity that can be achieved and safety.

**Table 4:** Typical questions PWH may have before deciding to enter a GT trial

Question
Which trial should I participate in?
What are the results, if any, from earlier phases of the trial?
What is the reputation of the trial team?
What vector is being used and what is the prevalence of pre‐existing vector antibodies?
Will pre‐existing antibodies automatically rule out trial participation or have strategies been developed to address this issue?
What vector dose is being infused and what is the anticipated range of factor expression? Is a higher vector dose worthwhile if the objective is higher factor expression?
Am I comfortable taking a prophylactic course of steroids if that is part of the protocol?
What duration of transgene expression is expected? What is the lower limit of duration of expression which would be persuasive to you in agreeing to participate in a trial or treatment? While lifetime expression is desirable, would I agree to treatment if expression was for 10 y? What about 1 y?
What is the potential for integration with an AAV vector? What is the likelihood of insertional mutagenesis and the risk of developing cancer in the future?
Is there a risk of inhibitor development?
Am I comfortable with the degree of monitoring and commitment required, especially in the first year, and with annual follow‐up for up to 15 y?

### 3.3 Questions regarding trial participation

In terms of the physician perspective, setting patient expectations, both for clinical trials and ultimately for gene therapy as an approved treatment option for haemophilia, will be important, as some PWH will not qualify for treatment. The standard inclusion/exclusion criteria employed in clinical trials to date have limited participation to adult patients with severe or moderately severe haemophilia,^33^, ^36^, ^37^ with exposure to factor treatment for defined periods of time (≥50 days minimum in published trials), and for most trials, normal liver and kidney function including absence of liver fibrosis. Trials generally exclude those with inhibitors, which would include approximately one‐third of people with haemophilia A. All of the published phase 1/2 studies for haemophilia A and B excluded patients with active hepatitis B or C (generally defined as active hepatitis antigen, DNA positivity or RNA viral load positivity) and active HIV infection (generally defined as positive serological test for HIV plus a CD4 T‐cell count of ≤200 per μL and detectable HIV viral load), although one study also excluded patients who were HIV positive.^37^ To date, all trials have also excluded patients with pre‐existing antibodies to the AAV serotype specific to each of the investigational products; however, at least one phase 3 trial for haemophilia B [NCT03569891] has lifted that exclusion criterion due to lack of evidence for associated reduced efficacy or immune responses due to pre‐existing low‐titre neutralising antibodies to AAV5.^45^ Exclusion of key populations such children and adolescents, women with haemophilia,^46^ and those with a history of inhibitors to factor replacement is consistent across Phase 1‐3 haemophilia GT trials, but a GT trial in people with haemophilia A and inhibitors has been announced.^47^ If GT does become available in children and adolescents, there may be ethical questions in terms of gaining informed consent. There has been an initial report of an adverse event potentially associated with a concomitant anti‐HIV drug,^48^ so if these kind of interactions are confirmed, care may need to be taken in treating some individuals with GT.

Beyond these factors, there are currently no characteristics that can be used to identify those who are likely to respond better or worse to GT, although this will likely become clearer as the evidence base grows. Importantly, the majority of newly approved treatments typically will likely gain indications specific to the populations studied in the clinical trials; thus, it will be important to set expectations for excluded populations on timeline of treatment availability and the necessity to gather evidence in these groups once GT becomes more established.

For patients who participate in clinical trials, there is a practical burden of frequent study visits in the short‐term, as well as long‐term (5 years on average) follow‐up, which may be underestimated by potential participants. A typical trial may require weekly or up to tri‐weekly visits in the first 6 months, monthly or quarterly visits up to 12 months and quarterly or bi‐annual visits for the remaining follow‐up despite participants potentially having normal or near‐normal factor levels. Participants will generally need to record factor use and bleeds using an e‐diary or similar approach, which will then be reviewed at each visit. During visits, body fluid samples will be required for vector shedding analysis; blood will be required for determining factor activity, inhibitors, liver enzymes, anti‐factor antibodies, or AAV antibodies/neutralising antibodies, inflammatory markers, T‐cell responses and other trial outcomes. Given that trials usually take place in specialist centres, study‐related visits will likely involve travel and potentially overnight accommodation. Other potential logistical issues include limitations on travel, the need for abstinence from alcohol in some trials, and potentially limitations on physical exercise to avoid muscle‐related transaminitis elevations. Even when GT is approved, it is likely that treated patients will need additional follow‐up to confirm sustainable transgene expression, clinical efficacy and safety.

From a patient perspective, with so many trials recruiting and ongoing, it is worthwhile to review the key outcomes and the types of questions PWH might have (Table 4). As discussed in the previous section, key outcomes for PWH include factor level, duration of factor expression, reduction in chronic pain, healthcare resource utilisation, impact on mental health and bleed frequency.^35^ Therefore, it is likely that individuals will choose to participate in trials based on the factors which are most important to them.

In an ideal scenario, the consent process should involve an independent person separate from the haemophilia treatment centre team who will clearly set out the potential risks to ensure full informed consent; however, this is not a requirement for consent in most trials. When introducing trials to PWH, it makes sense for initial communications to take place in small groups as such meetings often develop into discussion forums, which prompts questions that some attendees may not have thought about. Individuals should also familiarise themselves with relevant information from their national, regional or global haemophilia patient organisations. As GT becomes more established, it will be important for these organisations to provide patient friendly educational materials including video and to facilitate education sessions/lectures/conferences for their members. PWH should familiarise themselves with the trial protocol.

### 3.4 Sources of information on gene therapy

Given the gaps in the evidence base, it will be important to embrace a shared decision‐making approach.^49^ Physicians should give patients a clear understanding of the benefits and risks of GT based on the best available evidence at the time to enable a collaborative decision on the best treatment choice based on an individual's clinical history, preferences and treatment goals.^49^ As discussed below, patient organisations are also likely to be an important source of information. Connecting prospective trial participants with those who have received GT will also be invaluable, particularly in terms of the practicalities of treatment administration and what to expect following treatment. This ‘peer mentoring’ approach could be facilitated locally on a centre‐by‐centre basis or by patient organisations.

There are currently limited sources of high‐quality, independent information on GT. The National Hemophilia Foundation includes brief patient‐focused information on GT (https://www.hemophilia.org/Bleeding-Disorders/Future-Therapies) and details a free telephone number that can provide more information on novel therapies. The EHC (http://www.ehc.eu) launched a provider‐focused educational activity in collaboration with Medscape and a Novel Products newsletter that will be updated on a regular basis which covers GT in some detail.^50^ The EHC has released a series of five educational videos on GT designed for PWH. The World Federation of Hemophilia (https://www.wfh.org) has an online video covering several new treatment options including GT approaches. The American Society of Gene and Cell Therapy has two online webinars aimed at PWH, which provide an overview of GT in general and the role of GT in haemophilia (https://www.asgct.org/meetings-workshops/upcoming-webinars/hemophilia-webinars). The National Organization for Rare Diseases (https://rarediseases.org/rare-diseases/hemophilia-b/) has information on earlier GT trials.^21^, ^22^ Therefore, there is a need to provide a centralised, accessible and unbiased information source, so that PWH can access clear and easy to understand information on the novel therapeutic options in haemophilia.

### 3.5 The future of haemophilia gene therapy

It is an exciting time in GT, when the long‐heralded promise is starting to yield treatments that are entering the clinic. Most ongoing haemophilia GT trials utilise the process of gene addition, that is, infusing a healthy copy of a clotting factor gene (VIII or IX) via an AAV vector into a patient without altering their own DNA. There is at least one trial exploring gene editing for haemophilia, a process by which a zinc finger nuclease (sometimes referred to as ‘DNA scissors’) is used to insert the therapeutic transgene into a so‐called safe harbour or area with high‐transcriptional activity.^51^ Other types of GT are also being explored and it will be important for physicians to educate patients and families on the different options and discuss which approaches meet individual needs. It is likely that when GT initially become available that they will be prescribed through a limited number of expert centres, which should be fully able to discuss and educate patients about treatment options.

As haemophilia GT enters the clinic and is subject to surveillance, the longer‐term safety and efficacy profiles will become clearer. Theoretically, at least, it would make sense to initiate GT before joint damage is manifest, which may start between 1 and 2 years of age.^52^ While current trials in haemophilia are confined to adults, as the safety profile of GTs becomes more established, it will be important to include adolescents and children, so that treatments can be opened up to this important population. Treatment in younger populations, however, may present additional challenges in terms of the potential impacts of hormonal and developmental changes as well as liver growth on long‐term GT effectiveness. The development of inhibitors is a major problem that limits clotting factor treatment options and efficacy, so it will also be of great interest whether GT, either alone or when combined with other approaches such as immune tolerance induction, can benefit such patients, and the future results from the recently announced GT trial in haemophilia A with inhibitors will be awaited with interest.^47^ A number of different approaches have been studied in animals including classical immune tolerance induction with repeated exposure to antigens to therapies specifically targeting T or B cells.^53^

While the treatment of haemophilia has improved, it is costly and burdensome. Despite CFC, haemophilia still has major adverse impacts on the QoL of PWH including functional impairment, pain, and psychosocial issues. Longer‐term evidence is needed to confirm whether haemophilia GT offers durable efficacy precluding the need for factor replacement. Experience from clinical trials so far suggests that it offers a life‐changing opportunity for PWH to reduce their bleeding risk while also reducing or abrogating the need for exogenous factor administration.

## DISCLOSURES

W. Miesbach has received consultant fees from uniQure BV, grants and personal fees from Novo‐Nordisk and personal fees from Bayer, Shire, Biotest, Pfizer, Octapharma, LFB, CSL Behring, SOBI, Biogen, and BPL. B. O'Mahony declares no conflict of interest. N.S. Key has acted in the capacity of a consultant for uniQure BV and Spark Therapeutics. BV. M. Makris has participated in advisory groups for Freeline Therapeutics and Spark Therapeutics.
